# Effects of Pumice-Based Porous Material on Hydration Characteristics and Persistent Shrinkage of Ultra-High Performance Concrete (UHPC)

**DOI:** 10.3390/ma12010011

**Published:** 2018-12-20

**Authors:** Kaizhi Liu, Rui Yu, Zhonghe Shui, Xiaosheng Li, Xuan Ling, Wenhao He, Shuangqin Yi, Shuo Wu

**Affiliations:** 1 State Key Laboratory of Silicate Materials for Architectures, Wuhan University of Technology, Wuhan 430070, China; liu03303116@126.com (K.L.); zhshui@whut.edu.cn (Z.S.); li-xiaosheng@whut.edu.cn (X.L.); lingxuan@whut.edu.cn (X.L.); wushuo.phyche@hotmail.com (S.W.); 2 School of Materials Science and Engineering, Wuhan University of Technology, Wuhan 430070, China; 15730325018@163.com (W.H.); x3463973524@163.com (S.Y.); 3 Wuhan University of Technology Advanced Engineering Technology Research Institute of Zhongshan, Zhongshan 528437, China

**Keywords:** Ultra-High Performance Concrete (UHPC), long-term drying shrinkage, hydration characteristic, porous pumice, optimization

## Abstract

In this paper, two kinds of pumice particles with different diameters and water absorption rates are employed to substitute the corresponding size of river sands by volume fraction, and their effects on the hydration characteristics and persistent shrinkage of Ultra-High Performance Concrete (UHPC) are investigated. The obtained experimental results show that adopting a low dosage of 0.6–1.25 mm saturated pumice as the internal curing agent in UHPC can effectively retract the persistent shrinkage deformation of concrete without a decrease of strength. Heat flow calorimetry results demonstrate that the additional water has a retarding effect and promotes the hydration process. X-ray Diffraction (XRD) and Differential Thermal Gravimetry (DTG) are utilized to quantify the Ca(OH)_2_ content in the hardened paste, which can confirm that the external moisture could accelerate the early cement hydration and secondary hydration of active mineral admixtures. The Ca/Si ratio of C–S–H calculated by the Energy Dispersive Spectrometer (EDS) reveals that the incorporation of wet pumice can transform the composition and structure of hydration products in its effective area.

## 1. Introduction

UHPC (Ultra-high performance concrete), as a new type of cement-based material, is designed based on the principle of the closest packing between particles of each component with a very low water-to-binder ratio (w/b < 0.2) and a certain amount of reinforcing fibers [1,2,3,4]. Therefore, UHPC cement-based materials show great potential in applications which satisfy the requirements of structural engineering at a higher height, larger span, and heavier load [5,6,7,8,9,10,11]. Along with its excellent mechanical properties and durability, a strong continuous shrinkage deformation of the UHPC matrix can negatively affect its stability and reliability during the practical service life. This can be attributed to a rapid decrease in the relative humidity within the pores inside the UHPC system during the hydration process of cement at a relatively low w/b [12,13,14]. Therefore, how to effectively settle the problem of the large volume contraction of UHPC has become a hot topic in current research.

The accepted means to minimize the shrinkage of UHPC by employing internal curing carrier materials [15,16,17,18,19], expansive agents [20,21], shrinkage reducing agents [18,22,23], and other methods [24,25,26], can restrict the constriction development, especially its early self-shrinkage. However, the reduction of shrinkage by using an expansive agent alone is not recommendable due to its challenge in controlling the amount of swelling and occurrence time. A shrinkage reducing agent is often accompanied by the inhibition of early strength development and further deterioration of mechanical properties, if it has poor compatibility with chemical admixtures [27]. In contrast, an internal curing (IC) mechanism is considered to be a reasonable solution to fundamentally and effectively control UHPC contraction. Nowadays, most IC agents utilized in UHPC are super absorbent polymers and various types of lightweight aggregates (LWAs). It has been revealed that super absorbent polymers can significantly bate the growth of autogenous shrinkage of UHPC, and even fully compensate for the early contraction of concrete [15,28,29]. Nevertheless, the introduction of super absorbent polymers will negatively influence the compactness of UHPC and increase the porosity, resulting in degradation of the mechanical properties and durability of concrete, which are both important indicators of UHPC. Pre-wetting LWA is a kind of potential IC carrier material that can also delay the self-desiccation of UHPC through the slow-release effect of water, which has excellent volume stability during water absorption and release. Hence, the pre-wetting LWA can be potentially treated as an effective material to minimize the UHPC shrinkage based on experimental and practical points of view.

The commonly applied LWAs in concrete as IC agents include expanded clay, perlite, shale, other types of ceramsite, tuff, and pumice, etc. Philleo’s experiment found that the pre-wetted porous ceramic pellets could effectively reduce the self-shrinkage deformation of concrete, and explicitly put forward the concept of IC for the first time [30]. Bentz et al. [31,32,33] confirmed that the moisture introduced by LWA could not only restrain the contraction and cracking of concrete, but also improve the hydration degree of the surrounding mortar and intensify the strength. Besides, they proposed the formula for calculating the minimum amount of LWA for IC. Bentur et al. [34] showed that there was a significant correlation between the evolution of shrinkage performance and water content of pre-wetting ceramsite in high strength concrete. They found that the saturated ceramsite replacing ordinary aggregates by a volume fraction of 25% would completely offset the shrinkage and even swell. Akcay et al. and Henkensiefken et al. [35,36,37] demonstrated that the size of the aqueous LWA particles has an obvious influence on the effective range and efficiency of IC, and smaller granules were preferred. Henkensiefken et al. and Lura et al. [38,39] suggested increasing the substitution rate of soaked LWA, which could improve the relative humidity inside the paste and postpone the self-desiccation. Zhutovsky et al. [40,41,42] scientifically described the effect of exploiting pumice as the IC agent in high strength concrete. They showed that there was an optimal size distribution range of pumice particles in high strength concrete, and oversized or undersized particles would weaken the IC effect. The influence of incorporating saturated pumice on the evolution of total contraction deformation of high strength concrete within 100 days was related to the w/b of the system, while lowering w/b was beneficial to the dry shrinkage. Moreover, when adding wet pumice, the extent of hydration and total porosity of concrete increased, which might lead to impairing the characteristics of the densification matrix. Other scholars [43,44,45,46,47] also expressed concern about the potential negative effects of the introduction of water-absorbing LWAs on the strength, durability, and contraction distortion of concrete in a drying environment. In particular, RILEM TC 196-ICC [48] emphasized that adequate attention should be paid to the continuous shrinkage of IC concrete under dry conditions.

At present, there are few studies on LWAs as the IC carrier materials of UHPC. Suzuki et al. [49] claimed that using a porous ceramic-coarse aggregate as the IC agent of UHPC (w/b = 0.15) could slightly improve the compressive strength of concrete and depress autogenous shrinkage strain, and the effect on drying shrinkage was negligible. Van et al. [17] expounded that the moisture provided by the humid rice husk ash could not only delay the start-up time of the relative humidity drop inside UHPC, but also promote the continuous hydration of the cementitious system, thereby improving the mechanical properties and volume stability of concrete. The results of Wang et al. [50] showed that the incorporation of wet coral sand would clearly condense the self-shrinkage of UHPC, and the total contraction within seven days decreased by 53.9%. Meanwhile, there was a negative correlation between the development of concrete strength and the level of substitution. Meng and Khayat. [19] superseded river sand by volume fraction with 25% pre-saturated expanded shale, which could reduce the autogenous shrinkage deformation of UHPC by 25.4% and increase the compressive strength by 21.5% in 28 days. Nevertheless, the current concerns on the IC effect of LWAs on UHPC shrinkage are more focused on the early age (first 7 d or most up to 28 d), and the possible adverse effects from extraneous water brought in by the LWAs on the long-term persistent shrinkage deformation behavior under drying condition are usually ignored. In addition, the investigations regarding the effect of the gradation of LWA particles and content of extra water on the hydration process and hydration products in UHPC are limited.

Consequently, in this study, a typical LWA (pumice) is included in the production of UHPC, and the effects of extra water (from pre-wetting LWA) on the macro behaviors, including contraction continuous evolution under low humidity surroundings and the hydration of the composites cementitious system in UHPC, are investigated. The working performance, mechanical properties, and dry shrinkage of the developed UHPC are measured by standardized test methods, while microscopic analysis as hydration heat, XRD, DTG, and EDS are used to clarify some hydration characteristics.

## 2. Materials and Methods

### 2.1. Materials

OPC 52.5 cement (Huaxin Cement Co., Ltd., Huangshi, China), first grade fly ash (FA) (Huaneng Yangluo Power Plant, Wuhan, China), and silica fume (SF) (Southeast Star Technology Development Co., Ltd., Chengdu, China) are used as the active powders in this work and the XRF analysis results are shown in Table 1. Continuously graded river sand (Wuhan, China) and pumice particles (Changbai Mountain, China) (0–0.6 mm and 0.6–1.25 mm) are added as fine aggregates. A highly effective polycarboxylate super plasticizer (SP) (Sobute New Materials Co., Ltd., Nanjing, China), which has a solid content of 20% and a water reduction rate of 40%, is also added.

**Figure 1:**
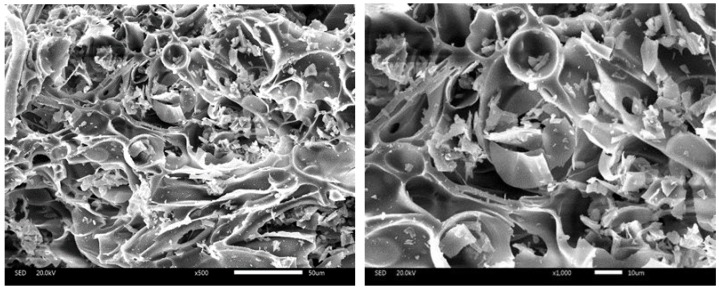
ESEM photomicrographs of the employed pumice stone.

**Figure 2:**
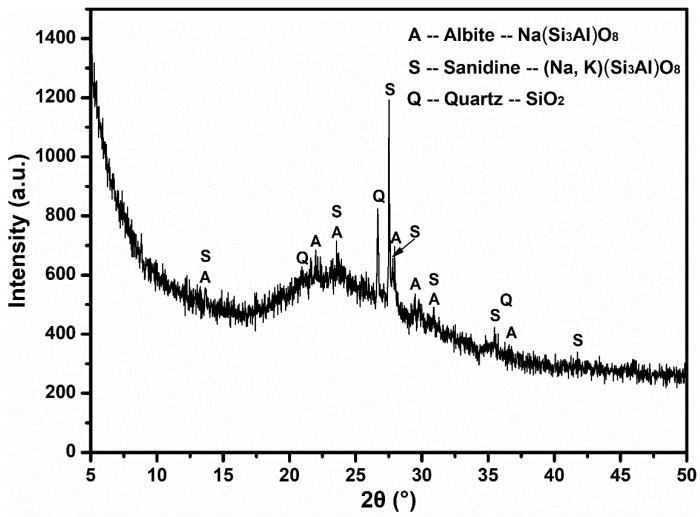
XRD pattern of natural pumice stone.

Pumice stone is well-known as a natural porous mineral material with a well-developed pore structure and an environment scanning electron microscope (ESEM) (FEI, Hillsboro, OR, USA) photomicrograph of it is shown in Figure 1. Pumice has a high porosity (more than 50%) and its pore framework is dominated by micron-sized slender tubular channels, and almost all of them are interconnected [35,51,52,53,54]. This typical conformation determines that it can be exploited as an efficient IC vector. The mineral phases are analyzed by XRD (Bruker, Karlsruhe, Germany) and the results are presented in Figure 2, which reveals that the main structure of pumice is Na+, K+ type amorphous phase feldspar with a brilliant stability (A: PDF (ICDD) 10-0393, S: PDF (ICDD) 10-0357, Q: PDF (ICDD) 46-1045). The main chemical components of pumice are SiO_2_ 70.55%, Al_2_O_3_ 11.21%, Na_2_O 4.79%, Fe_2_O_3_ 4.57%, K_2_O 3.76%, and CaO 0.81%, which are provided by XRF. Meanwhile, the apparent densities of 0–0.6 mm and 0.6–1.25 mm pumice are 1.08 g/cm^3^ and 0.59 g/cm^3^, respectively, and the corresponding saturated water absorption rates of them are 77.9% and 66.6%, as determined by the tissue wiping method [55,56].

### 2.2. Experimental Methodology

#### 2.2.1. Design Method

The revised Andreasen-Andersen (A&A) model is widely applied in the optimization design of the UHPC mix ratio. Studies confirmed that the optimal range of the distribution coefficient (q) is 0.22~0.25 [50,57,58], and the value of *q* is selected as 0.23 in this paper.

In order to retain the accumulation state of the UHPC system, the pumice replaces the same-grain river sand with a certain volume ratio. According to the measured particle size distribution of the active powders and the two fine aggregates, the method of least squares (Equation (1)) is used to evaluate the close packing state of granules in UHPC, and the reasonable mixture ratio design scheme of the matrix is exhibited in Table 2, where C0 represents the control group; U and S indicate the water absorption state of the pumice, where U represents unsaturation and S represents saturation; and P_1_ and P_2_ represent the 0–0.6 mm and 0.6–1.25 mm pumice particle substitution system, respectively.

(1) $$RSS=\frac{\sum_{i-1}^{n}{\left(P_{mix}\left(D_{i}^{i+1}\right)-P_{tar}\left(D_{i}^{i+1}\right)\right)}^{2}}{n}$$

where $D_{i}^{i+1}$, *n*, *P_mix_*, and *P_tar_* denote some gradation of particle size (μm), the number of chosen particle sizes, the designed mix, and the target curve, respectively. RSS is sum of the squares of the residuals, which represents the proximity of the designed mix and target curve.

**Figure 3:**
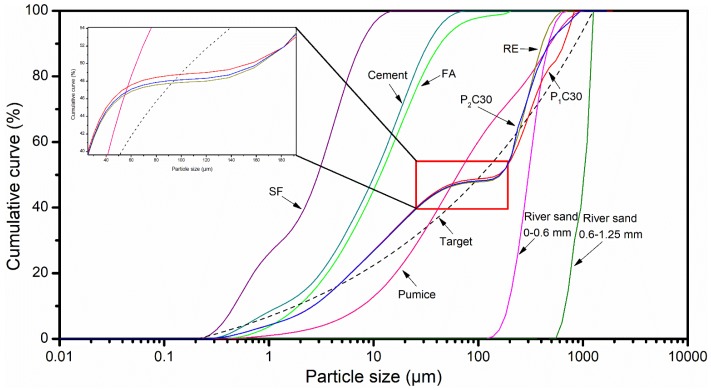
Particle size distributions (PSDs) of the crude materials, the target, and optimized grading curves of the composites on account of optimized pre-wetting pumice substances.

The closest stacking curves of the raw materials emerged in Figure 3. It is shown that although the gradation of pumice particles in the same size range is different from that of river sands, no obvious deterioration of the accumulation state between the particles can be observed, whose form are still in a tight status.

#### 2.2.2. Flowability

The fluidity of the fresh UHPC paste is evaluated by GB/T 2419-2005 [59]. The test die includes a truncated cone mold and a pattern sleeve, wherein the mould size is as follows: the inner diameters of the upper and lower ports are 70 mm and 100 mm, respectively; and the outer diameter of the lower mouth and height are 120 mm and 60 mm, respectively.

#### 2.2.3. Mechanical Properties

UHPC compressive and flexural strength is tested according to GB/T 17671-1999 [60]. The sample size is 40 mm × 40 mm × 160 mm, and the samples are kept in water at a temperature of 20 ± 2 °C. In the experiment, six specimens per mix-age are tested.

#### 2.2.4. Drying Shrinkage

The measurement of UHPC drying shrinkage is based on JC/T 603-2004 [61]. Each group is formed of three samples of 25 mm × 25 mm × 280 mm, and the test blocks is kept in 20 ± 2 °C water for seven days after demolding. After maintenance, the initial length of the specimens is recorded as L_0_, and the pieces are transferred into the dry shrinkage curing box with a condition of temperature of 20 ± 2 °C and humidity of 60 ± 2% as the “time zero”, and the length L*x* is measured at the corresponding time.

#### 2.2.5. Hydration Process

To evaluate the effect of extra water introduced by the pre-wetting pumice on the cement hydration kinetic in the UHPC composite system, a heat flow calorimetry modeled TAM AIR is employed. The paste is strictly designed according to the recipes; simultaneously, the rate of heat liberation and total heat within 72 h are monitored.

#### 2.2.6. XRD

The hydration products of UHPC are characterized by a D8 Advance diffractometer with Cu Kα radiation, a step size of 0.019 °2θ/step, a measuring time of 141.804 s/step, a start position of 5°(2θ), and an end position of 50°(2θ).

#### 2.2.7. DTG

The standard ground sample powders are subjected to thermogravimetric analysis using a NETZSCH STA 2500 synchronous thermal analyzer at a heating rate of 10 °C/min up to 1000 °C.

#### 2.2.8. ESEM-EDS

The micro-domain morphology and structure of the samples’ surface are observed by a QUANTA FEG 450 field emission ESEM. At the same time, the EDS analysis can interpret the micro-area components, and the intelligent quantitative results provided by eZAF help to effectively evaluate the atomic ratio of the skin layer elements.

## 3. Results and Discussion

### 3.1. Fresh Behaviors

The relationship between the fluidity of the fresh paste and the amount of extra water introduced under different mixing ratios is shown in Table 3. The given results indicate that under the same substitution ratio, there is a good positive correlation between the liquidity and the content of water imported; that is, the more water that is introduced, the greater the flowability of the paste. This is attributed to the increase of internal moisture in UHPC, which means that more water participates in the lubrication of the particles, resulting in the increase of the fluidity of the newly poured mortar. Compared with the behavior of the control group, the liquidity of the UHPC compound system is determined by the role of wet pumice particles (whether continuously injected water into the paste or siphon free moisture from the paste during the mixing process). As the water absorption of the pumice particles does not reach saturation, the mortar fluidity will only increase when the volume substitution fraction reaches 30%, whose liquidity exceeds 200 mm (C0 = 183 mm). When the replacement grade is less than 20%, the flowability drops to a minor extent, with a minimum of 165 mm. When the pumice pellets reach a water saturated state, the paste fluidity is controlled by the amount of water introduced. If the content of extra water exceeds 10 g, the mobility of the paste will improve, and the greater the amount of water introduced, the greater the fluidity of the new mixture. When the external water content reaches 72.9 g, the flow value reaches the maximum value of 374 mm, attaining 204.4% of the control group.

### 3.2. Mechanical Properties

**Figure 4:**
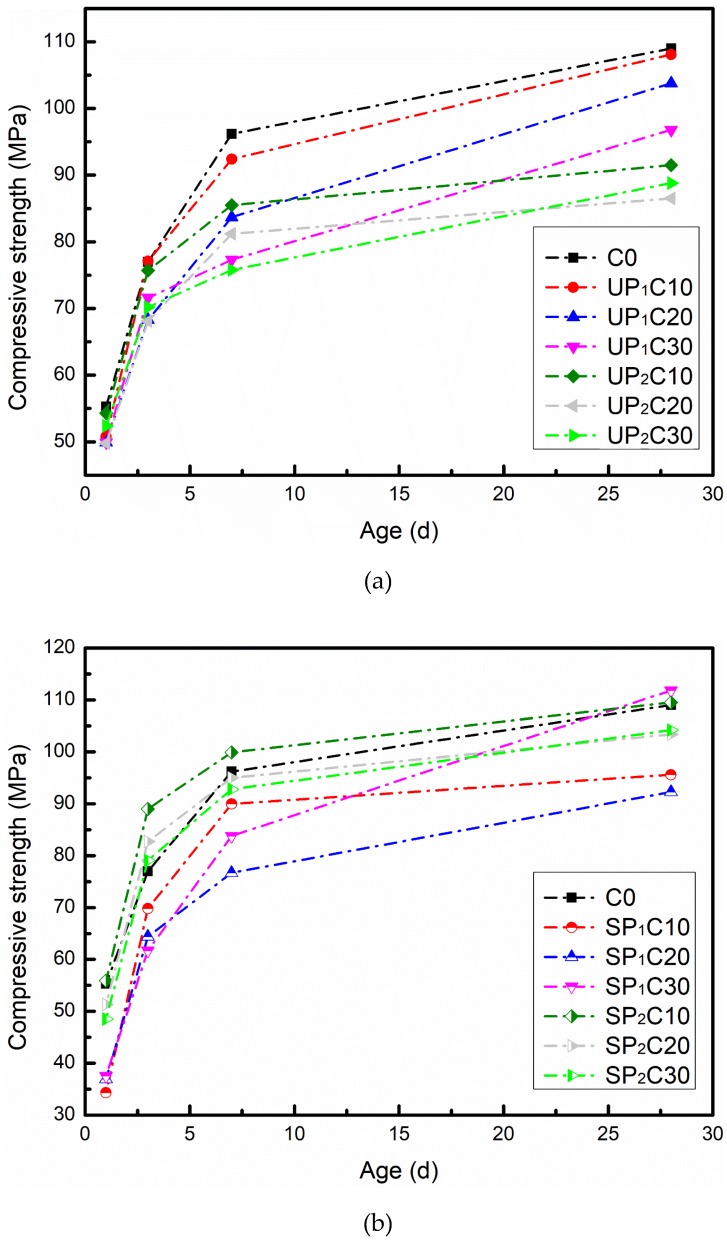
Compressive strength development of UHPC combined with water absorption pumice: (**a**) unsaturated pumice substitution; (**b**) saturated pumice substitution.

**Figure 5:**
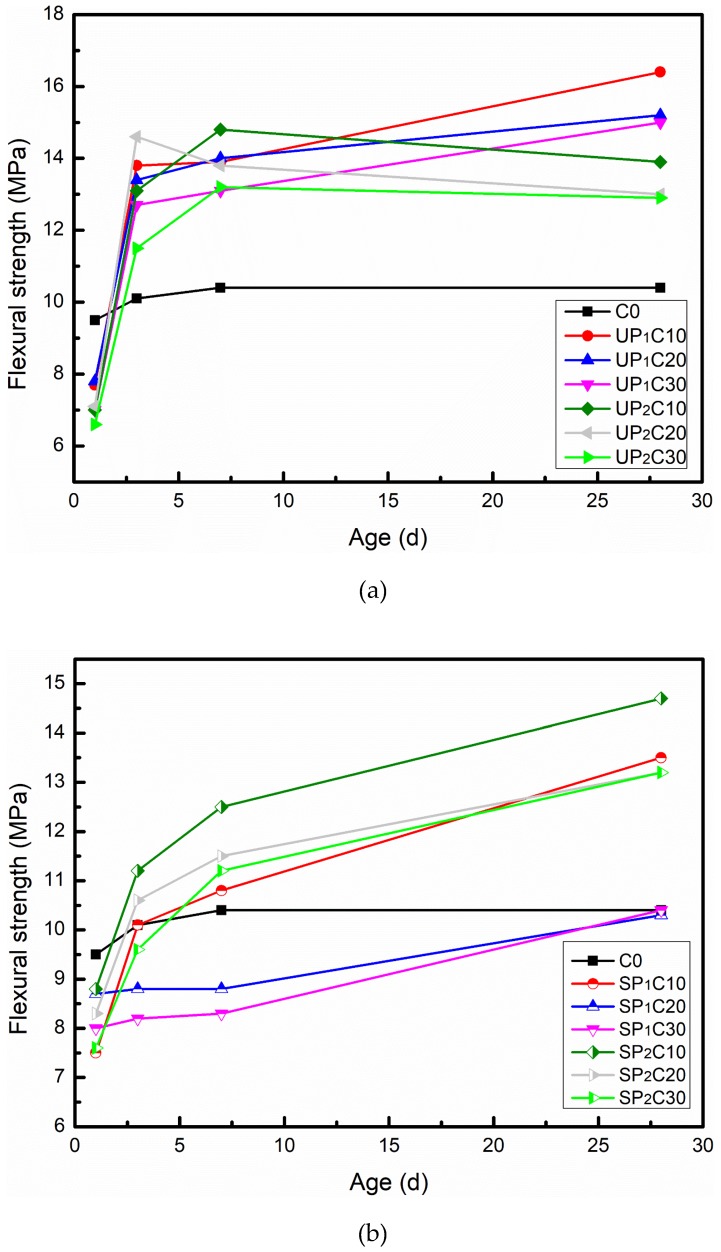
Flexural strength variation of UHPC combined with water absorption pumice: (**a**) unsaturated pumice substitution; (**b**) saturated pumice substitution.

The compressive and flexural strengths of the designed UHPC with different formulas are shown in Figure 4 and Figure 5, respectively. The compressive strength results show that the addition of soaked pumice reduces the compressive strength of UHPC after 28 d curing as a whole, and the average strength of each group is 91.2% of C0, while the maximum compressive strength loss rate (UP_2_C20) is 20.6%. Among them, the strength development of UP_1_ and UP_2_ substitution systems has a similar law; that is, the strength decreases as the content of extra water increases. The distinction is that the average 28 d compressive strength of UP_1_ groups is 5.6% lower than that of C0, while that of UP_2_ is 18.4%. For the groups of 0–0.6 mm pumice particles to replace the homologous river sands, as the content of introduced water is in the range of 17.7~48.6 g (UP_1_C30, SP_1_C10, and SP_1_C20), the compressive strength of each age has obvious loss. Meanwhile, the amount of extra water is less than 11.8 g (UP_1_C10, UP_1_C20) or up to 72.9 g (SP_1_C30), the compressive strength loss at 28 d is kept at a low level, and even a slight improvement can be observed. For that 0.6–1.25 mm pumice substitution system, the key factor determining the compressive strength of UHPC is the water absorption state of pumice. If the pumice granules do not reach the saturated water absorption, the strength is significantly depressed. The strength can be held at a similar gradation as the control group while the pumice particles are full of water.

The flexural strength results show that the flexural strengths of the SP_1_C20 and SP_1_C30 groups with a water content exceeding 48.6 g express a downward trend compared with the control group. In addition to the above both groups, the bending strength performance of other recipes embodies the same regular pattern, which is that the bending strength decreases with increasing extra water intake within a uniform substitution system, which remains notably higher than that of C0. The details can be inducted as follows: the flexural strength of the group blended with damp pumice after 1 d is significantly lower than that of C0, with an average value of 80.8%. It grows rapidly within 1~3 d and almost completely exceeds that of C0 at 3 d, with an average of 113.5%. By 28 d, the average bending strength of each group reaches 129.6% compared with C0, and the highest value achieves 157.7% (UP_1_C10).

The incorporation of pre-moistened pumice has both positive and negative effects on the development of UHPC mechanical properties. Among them, the positive effect is that the sustained release of moisture by the wet pumice will continuously promote the hydration of the unhydrated cement particles and secondary hydration of reactive powders (SF and FA) in the cementitious system and then enhance the gelling ability, whose contribution to the development of strength mainly affects the later stage. The negative effect comes from two aspects: primarily, the water released from the pumice pellets will cause the early actual w/b of the system to rise, thereby affecting the porosity and types of hydration products, which brings a reduction of strength [62]. Secondly, the elastic modulus and crushing value of the pumice particles are clearly lower than those of natural river sand, and the fine mineral aggregates are destroyed under the action of external pressure or impact force, which leads to failure of the specimen [52,54,63]. In a word, the characteristic development of mechanical properties of pumice-based UHPC is a reflection of which effect dominates.

### 3.3. Persistent Shrinkage Deformation

**Figure 6:**
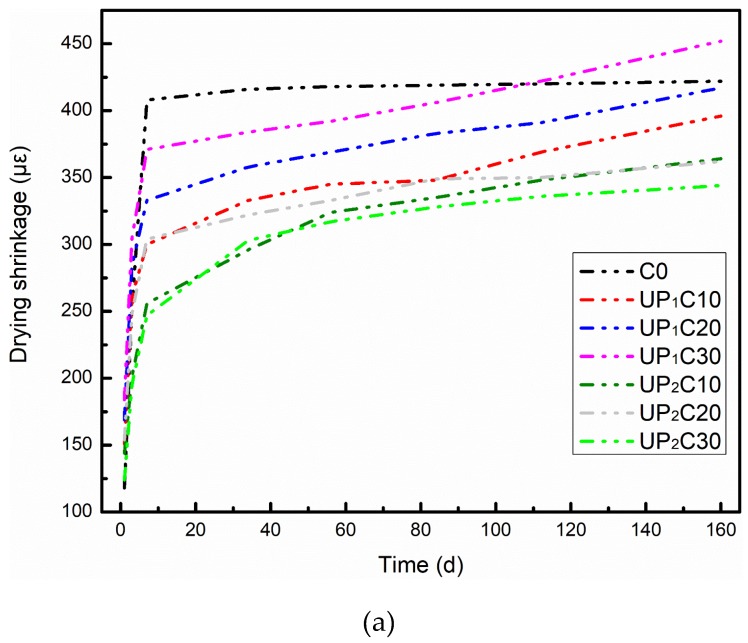

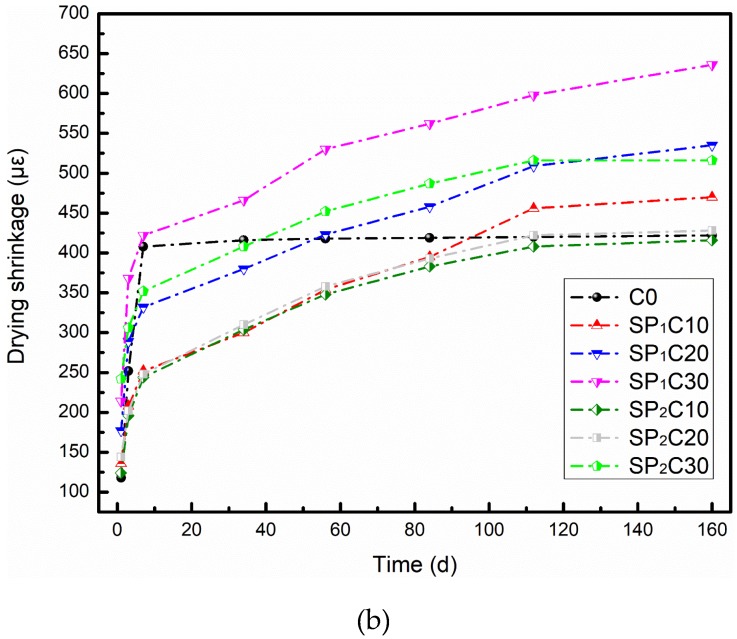
Influence of humid pumice on the persistent shrinkage deformation evolvement of the designed UHPC: (**a**) unsaturated pumice substitution; (**b**) saturated pumice substitution.

The drying shrinkage curves of UHPC designed with different mix ratios are presented in Figure 6. Actually, the trend of drying shrinkage development is the collective result of the combined action of autogenous shrinkage after seven days and continuous dry shrinkage deformation in the later low humidity environment of concrete. Thus, the curves reflect the sight of the total shrinkage. The given information is that the drying shrinkage evolution characteristics of the UHPC system compounding damp pumice are significantly different from those of the control group. The dry shrinkage develops with a uniform growth in C0 within seven days, and the contraction behavior basically disappears and reaches a volume stable state after that. The first seven days of shrinkage development of UHPC with damp pumice is also swift, but the rate then gradually decreases, and the test piece continues to contract within 7 d~160 d. The constriction development of concrete is related to the moisture migration and pore structure of concrete [64,65]. The w/b in UHPC is extremely low, and the moisture is quickly consumed in the early stage of hydration, resulting in a rapid decline in the relative humidity inside the system. Therefore, regardless of whether the concrete introduces an IC agent, its early (within 7 d) shrinkage will increase quickly. UHPC has a low porosity and dense architecture, which makes it difficult to exchange moisture with the outside surroundings. Consequently, the C0 group can maintain a relatively stable evolution state after rapid development in the early stage. After being mixed with the humid pumice, the water slow-release effect of the pumice “tank” can effectively delay the decline of the internal relative humidity. Besides, the pumice pore structure has the function of retaining moisture, and restrains the diffusion of water to the outside. The effects of both mentioned above are superimposed, and the rate of contraction development in the UHPC within 7 d of adding the wet pumice is reduced. The release of water from the pumice in the concrete is a continuous process, and the moisture will increase the actual w/b of the cementitious system, resulting in a raise in the total water capacity and porosity, which will elevate the permeability of UHPC [42]. At the same time, the pore structure of the porous pumice itself may also broaden the channels communicating with the external world. Thus, the drying shrinkage distortion of the UHPC combining the water-absorbing pumice will last for a long time.

The drying shrinkage of C0 after 1 d, 7 d, 34 d, and 160 d is 118 με, 408 με, 416 με, and 422 με, respectively. Compared with the control group, the contraction value of the designed UHPC with pre-wetted pumice at 1 d is increased, and the promotion of each compounding system is 28.0%, 43.2%, and 55.9% with UP_1_C10~UP_1_C30; UP_2_C10~UP_2_C30 is 22.0%, 30.5%, and 5.1%; SP_1_C10~SP_1_C30 is 15.3%, 50.0%, and 81.4%; and SP_2_C10~SP_2_C30 is 5.1%, 22.0%, and 105.1%, respectively. The analysis results indicate that the development of the shrinkage of the complex UHPC within 1 d is not directly related to the quantity of extra water, and the soaked pumice particles of 0.6–1.25 mm are more sensitive to drying shrinkage than those of 0–0.6 mm. The content of foreign water by SP_1_C10 is 24.3 g, and the constriction is increased by 15.3% compared with C0. Oppositely, the amount of water introduced by SP_2_C20 is 6.7 g, but the corresponding shrinkage value is increased by 22.0%. The extent to which the contraction characteristics of the concrete are affected is determined by the actual water participation in hydration that can effectively promote a reaction in the very early period. In fact, this process is related to the water release behavior of water-absorbing pumice grains in the interior of UHPC. The smaller the particle size of pumice after crushing, the lower the proportion of coarse holes and the connectivity in the pore structure [41]. It is generally believed that the water release behavior of the pore structure has a higher priority for the one with a larger diameter, which means that the larger beads have a faster water release rate and higher efficiency [66,67,68]. Under the same conditions, the size of 0.6–1.25 mm pumice granules can supply more water for the IC. In addition, the greater scale of the “miniature reservoir” that affects the effective area of hydration is more expansive [63,69,70].

In the 1~7 d age range, the shrinkage growth of the UHPC appending the humid pumice is still relatively fast, but its development rate begins to gradually slow down, and the total value of contraction within 7 d is lower than the control group. Compared with C0, the details of the total dry shrinkage rate during 7 d are that UP_1_C10~UP_1_C30 shrinks by 26.5%, 18.4%, and 9.1%; UP_2_C10~UP_2_C30 retracts by 37.3%, 25.5%, and 39.5%; SP_1_C10~SP_1_C30 shrinks by 38.2%, 18.6%, and −3.4%; and SP_2_C10~SP_2_C30 retracts by 40.2%, 39.2%, and 13.7%, respectively. It reveals that the intake of moisture at this stage remains the postponed effect of self-desiccation inside the matrix. The shrinkage of SP_1_C30 after 7 d has exceeded the value of C0, and it continues to grow in the later period at a high-speed. This can be attributed to the fact that the content of external water in the SP_1_C30 group is too large, the total w/b of the mortar has reached 0.25 (beyond the scope of UHPC), which is disadvantageous to the microstructure development, and the porosity of concrete is then broadened, resulting in the deformation due to continuous moisture loss increases.

After seven days of hydration, the drying shrinkage of UHPC with dank pumice adjunction continues to develop. The total drying shrinkage of each group at a 160 d age (C0 = 422 με) is 396 με, 417 με, and 452 με for UP_1_C10~UP_1_C30; 364 με, 362 με, and 344 με for UP_2_C10~UP_2_C30; 470 με, 535 με, and 636με for SP_1_C10~SP_1_C30; and 416 με, 428 με, and 516 με for SP_2_C10~SP_2_C30, respectively. Among them, in the 0–0.6 mm wet pumice particles replacement system, the extra water content more than 17.7 g will lead to an increase in the final shrinkage of UHPC. In that substitution group of the 0.6–1.25 mm pumice, the amount of water introduced will only need to be greater than 6.7 g, and the total contraction will rise. The regularity that can be summarized is that the long-term shrinkage of UHPC combined with wet pumice is positively correlated with the amount of water introduced, and the larger particles contribute more to the final contraction deformation. This phenomenon can also be due to the higher water release efficiency of the larger size of pumice particles.

### 3.4. Hydration Process Analysis

**Figure 7:**
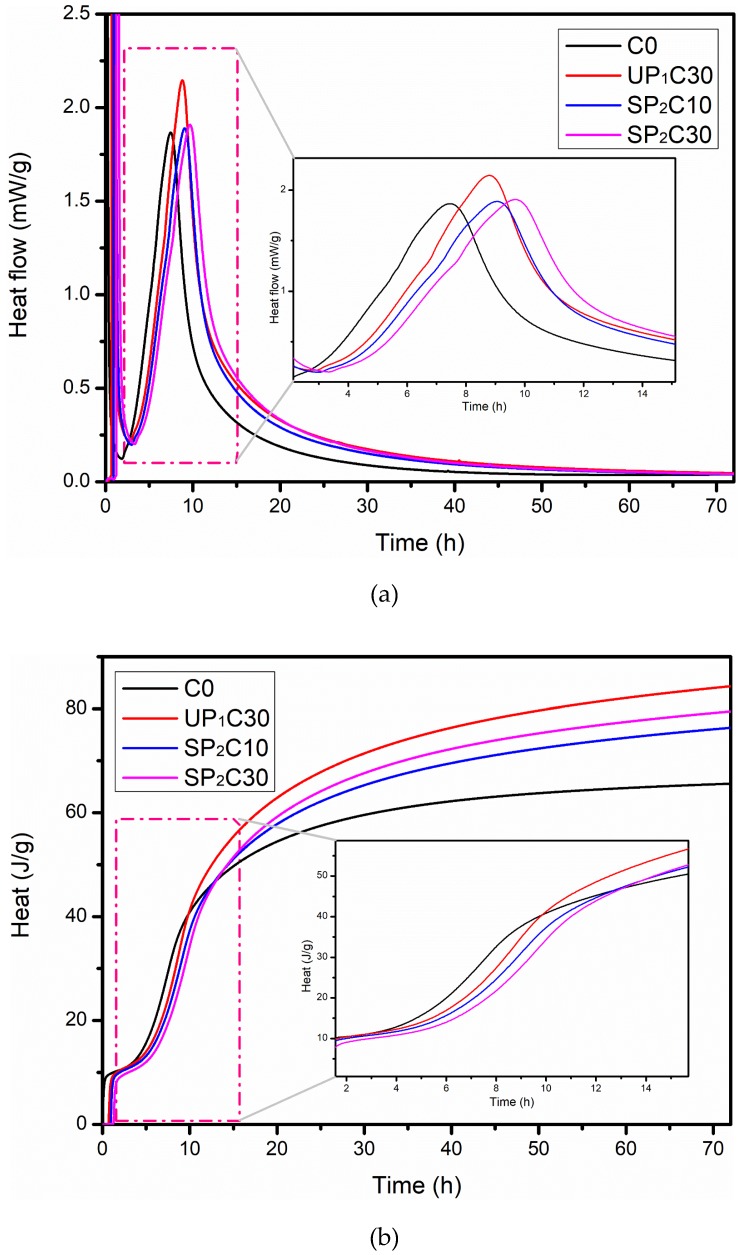
Heat evolution of UHPC system consisting of hydrated pumice within 72 h under several typical mix design conditions: (**a**) heat flow; (**b**) total heat.

The influence of extra water on the heat flow and total heat in the UHPC system within 72 h under several typical mix design conditions is collected in Figure 7. The results indicate that the external moisture introduced by cellular mineral will not change the basic law of cement hydration, but merely has an impact on the hydration course, including exothermic peak location and total heat output. Moreover, it mainly affects the very early hydration, and has a limited influence on the stabilization period. The heat flow curves indicate that C0, UP_1_C30, SP_2_C10, and SP_2_C30 all have a distinct hydration exothermic peak, and the distribution range is 2~20 h after mixing, with good consistency. Despite the same tendency being presented, there are divergences between the exothermic peak values and time schedules in the violent stage, possessing the similar rules. In the period of acceleration, the exothermic peak of C0, UP_1_C30, SP_2_C10, and SP_2_C30 is 1.87 mW/g, 2.15 mW/g, 1.89 mW/g, and 1.91 mW/g, respectively, and the corresponding occurrence time is 7.5 h, 8.8 h, 9.1 h, and 9.7 h. The height of the exothermic peak is positively correlated with the amount of extra water, which can persistently promote the progress during the hydration step [71]. The emergence time of that peak is related to the abundance of moisture in the vicinity of cement particles. Compared with the 0–0.6 mm pumice bead, the water-releasing rate of 0.6–1.25 mm is faster, which manifolds the gross of hydration products on the surface of cement granules within the effective range. The formation of the hydrated products film gravely inhibits the chemical reaction, leading to a modest lag in the time table for that crest [72]. The retarding effect of introducing water is an important reason for the significant decrease in the mechanical properties of UHPC mixed with wet pumice in one day.

The profiles of total heat reveal that the quantity of heat of C0, UP_1_C30, SP_2_C10, and SP_2_C30 within 72 h is 65.6 J/g, 84.3 J/g, 76.3 J/g, and 79.5 J/g, respectively. As can be gathered, the more added water there is, the higher heat the UHPC system produces. There is an environment with an extreme water shortage inside UHPC, and extra moisture will eventually participate in the reaction at different stages of hydration, improving the degree of hydration and the development of compensation strength.

### 3.5. XRD

**Figure 8:**
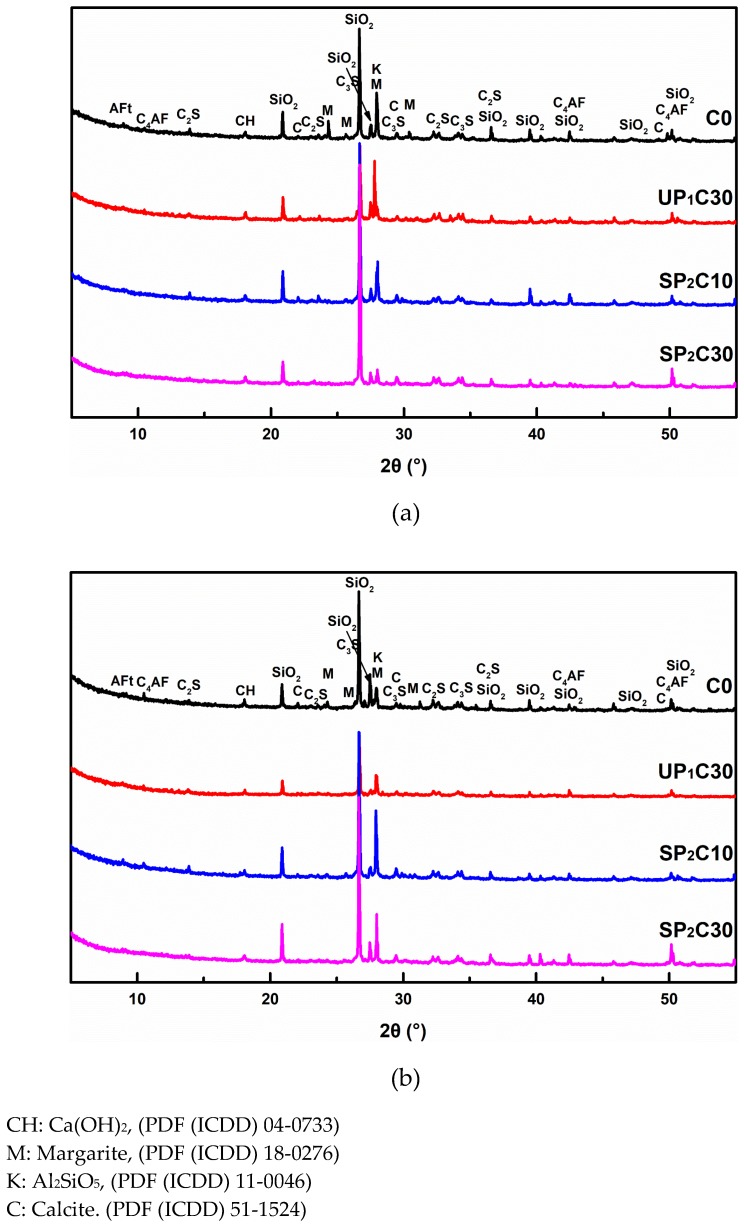
XRD pattern of UHPC hardened paste formed with pre-humid pumice at 7 d and 28 d age: (**a**) 7 d age; (**b**) 28 d age.

The XRD patterns of several representative UHPC samples at 7 d and 28 d are given in Figure 8. The characterizations of hydration products elaborate that the main phases of different ages in UHPC are Ca(OH)_2_, Aft, and CaCO_3_, and the introduction of water-absorbing pumice does not produce byproducts. Besides, there are obvious C_2_S, C_3_S, and C_4_AF characteristic peaks in each group due to unhydrated cement particles in the UHPC hardened paste.

**Figure 9:**
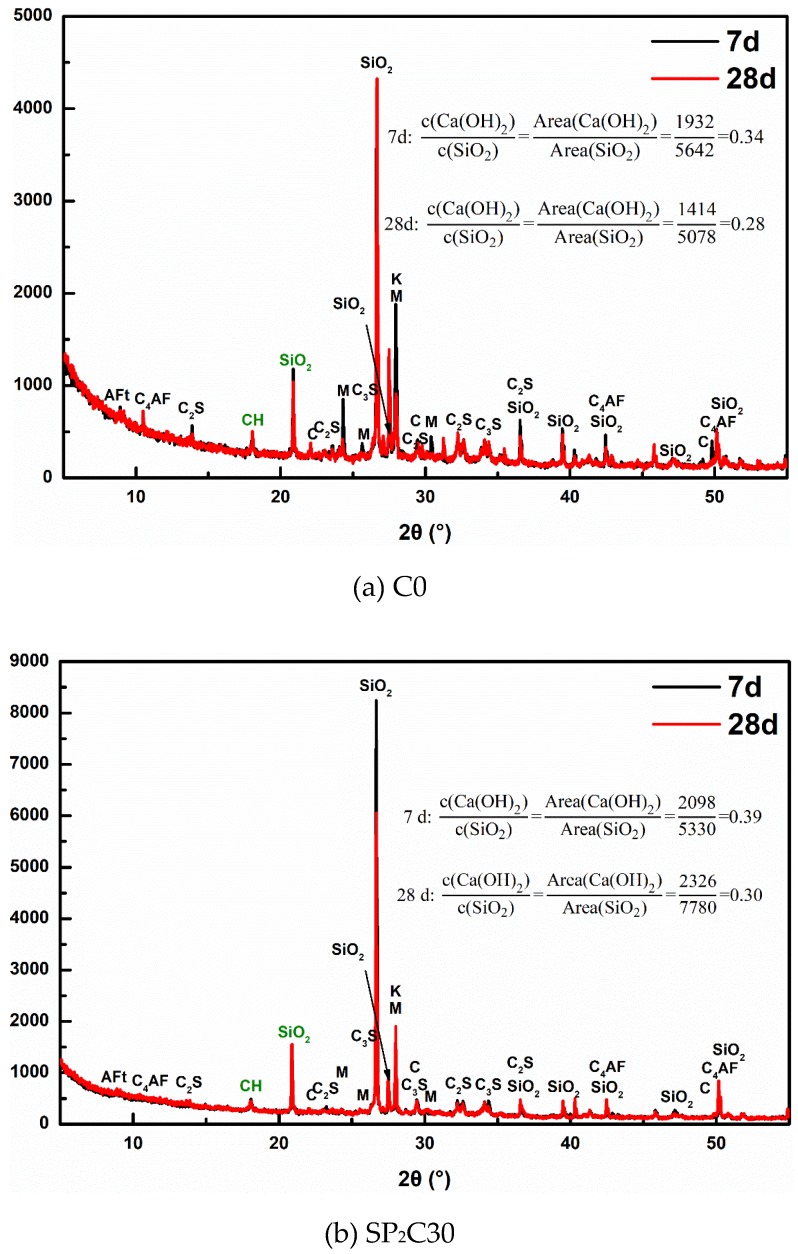
XRD qualitative analysis the content of Ca(OH)_2_ in specimens of C0 and SP_2_C30 groups for 7 d and 28 d curing age: (**a**) C0; (**b**) SP_2_C30.

To have a better understanding of the hydration mechanism of wet pumice in UHPC, the Ca(OH)_2_ content in the 7 d and 28 d samples of the SP_2_C30 group is qualitatively analyzed and calculated as shown in Figure 9. A specific SiO_2_ peak is chosen as the control object (2θ = 20.89) to furthest eliminate the fluctuation of test errors, and the ratio of Ca(OH)_2_ to the corresponding peak area of that SiO_2_ in diffraction patterns of the 7 d and 28 d concrete specimens is computed. Evaluation results reveal that the Ca(OH)_2_ consumption rate in C0 is 17.6% in the range of 7–28 days, while that of SP_2_C30 is 23.1%. It proves that the external moisture carried by the IC agent will stimulate the second hydration of pozzolanic active admixture in the system earlier, which is consistent with the results of hydration heat analysis. The introduction of external water to promote the hydration process of the UHPC cementitious system (Cement, FA, and SF) is an important source of compensation strength development.

### 3.6. DTG

**Figure 10:**
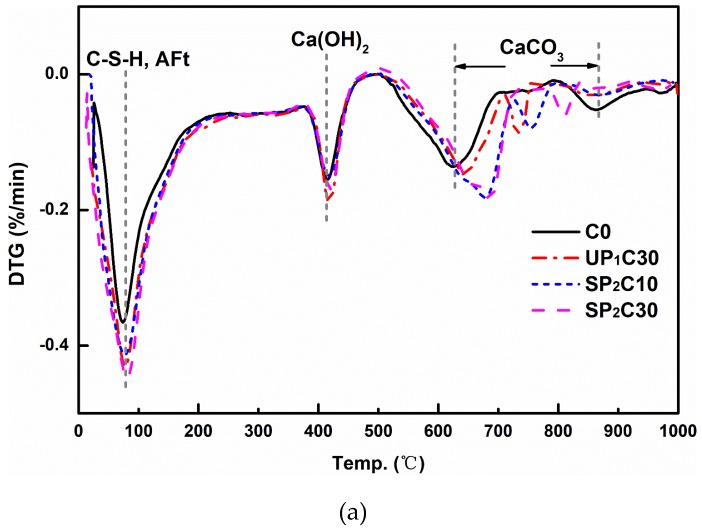

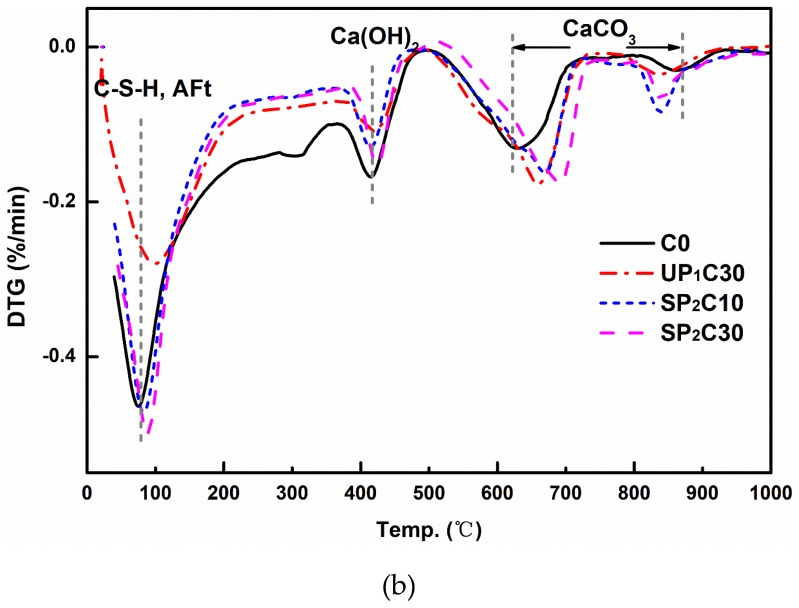
Effect of wet pumice on the thermal decomposition characteristics of several typical UHPC recipes after 7 d and 28 d: (**a**) 7 d; (**b**) 28 d.

Thermogravimetric analysis is also performed on the samples mentioned above, and the DTG curves for the tested specimen at 7 d and 28 d are exhibited in Figure 10; incidentally, the yield of Ca(OH)_2_ based on calculation is presented in Table 4. According to Figure 10a, the hydration of the cementitious system at 7 d is accelerated by incorporating wet pumice. The weight loss curve of each group at different stages is comparatively analyzed, and sums up that the UHPC designed with the IC mechanism compared to the control group has the following details: (1) The mass loss of C–S–H and AFt in the composite mortar within the range of 100~200 °C while the influence of adsorbed water has been excluded becomes larger, indicating that the content of hydration products in the gelling system increases; (2) although the total amount of CaCO_3_ formed by carbonation of Ca(OH)_2_ is significantly increased, the remaining quality of Ca(OH)_2_ is still large, testifying that the production of Ca(OH)_2_ is also palpably raised, which is sensitive for the large beads; (3) the location of the main decomposition peak of CaCO_3_ manifests some right shifts, which means that the carbonation time is prolonged, and the incipient gelation framework develops slowly. This phenomenon has been corroborated and supported by the widespread suppression of its 1 d strength development.

The results shown in Figure 10a,b have a good overall consistency, demonstrating that the effect of pumice containing water on the promotion of hydration of the UHPC system is a continuous process, and the reasoning agrees with the deduction of XRD analysis. After the introduction of the IC pumice, the content of Ca(OH)_2_ is notably lowered at a 28 d age compared to the 7 d age samples; contrarily, that of Ca(OH)_2_ is increased in the control group of UHPC. This is additional evidence that the extra water will promote the secondary hydration of SF and FA in the composite system, which consumes part of Ca(OH)_2_.

### 3.7. ESEM-EDS

**Figure 11:**
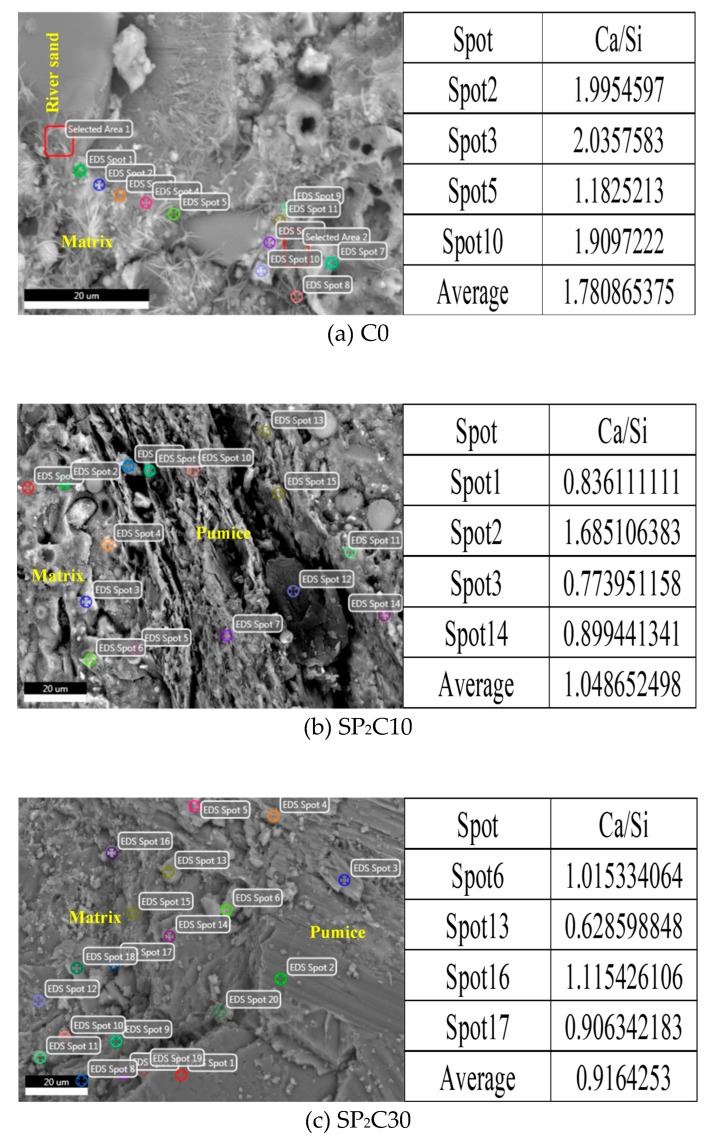
EDS elemental content analysis of the matrix nearby river sand and wet pumice in C0, SP_2_C10, and SP_2_C30 samples mentioned above after 28 d curing: (**a**) C0; (**b**) SP_2_C10; (**c**) SP_2_C30.

C–S–H gel is the most important hydration product and cementing component in concrete. It is generally accepted that the C–S–H in the hydration products of cement-based materials is divided into two types: C–S–H (I) and C–S–H (II). Among them, Ca/Si of C–S–H (I) is 0.6–1.5, and Ca/Si of C–S–H (II) is about 2.0 [73,74]. The Ca/Si of C–S–H in the hydration products of the 28-day-old UHPC specimen pieces is evaluated by ESEM-EDS, as exhibited in Figure 11. The results reveal that the introduction of humid fine aggregates will lead to a sharp drop in the average Ca/Si of C–S–H in the neighboring mortar. This means that the IC moisture can not only promote the hydration process of the UHPC gelling system, but also make it easier for the reaction to form C–S–H (I), which bears a lower degree of polymerization and crystallinity. The mechanism that causes this phenomenon comes from two aspects: firstly, water introduced by wet pumice will increase the actual w/b of the mortar within its effective range, resulting in a change in the regional hydration products, especially the C–S–H structure [75,76]. Secondly, the Ca/Si in C–S–H is proportional to the content of Ca(OH)_2_ in hardened paste [77], as mentioned in the previous section, and the IC moisture can activate the pozzolanic reaction of active admixtures more and consume Ca(OH)_2_, resulting in aCa/Si decrease in C–S–H. The mutation of the C–S–H structure has a non-negligible influence on the UHPC strength development and the extremely late drying shrinkage deformation using the IC mechanism, while C–S–H loses interlayer water and C–S–H gel particles produce irreversible permanent rearrangement [78,79].

## 4. Conclusions

In this paper, the effects of water-absorbing pumice on the persistent drying shrinkage and hydration characteristics of UHPC are investigated. Combined with comprehensive characterization analysis, the conclusions are summarized as follows:

(1) The flowability of UHPC fresh paste is related to the water absorption and release behavior of wet pumice during mixing. For the 0–0.6 mm humid pumice substitution system, the injection action plays a dominant role when the water content exceeds 17.7 g. For that of the 0.6–1.25 mm replacement system, the volume quantities need more than 20% to ensure that the injection action has priority.

(2) The introduction of IC water will result in an average decrease of 8.8% in the compressive strength of UHPC after 28 d curing; meanwhile, the flexural strength will be increased.

(3) Incorporation of impregnated pumice will inhibit the early drying shrinkage deformation behavior of UHPC, but the specimen will continue to contract during the later stage. There is a satisfied rule that the final total shrinkage is proportional to the content of extra water for the unified displacement system, while the larger size of LWA IC granules has greater potential adverse effects on the persistent dry contraction of concrete.

(4) The optimum recipe is 0.6–1.25 mm pumice particles in a saturated water absorption state replacing 10% river sands with the same particle size by volume fraction (SP_2_C10). Its compressive strength increases slightly and the flexural strength increases by 41.3% at 28 d. Meanwhile, the total shrinkage is effectively reduced within 160 d.

(5) The additional water has a retarding effect on mortar, which will delay the peak of the hydration exotherm, but raise the rate of heat release during the accelerated period of hydration and the total exothermic energy of the system.

(6) The introduction of water into pumice will promote the hydration process of the UHPC cementitious system. This effect is a continuous proceeding, including early cement hydration and secondary hydration of the reactive powders.

(7) The wet pumice will make the hydration reaction of the paste nearby more likely to generate C–S–H (I) with poor crystallinity, resulting in changes in the composition and structure of hydration products in its effective area.

## Figures and Tables

**Table 1:** Chemical compositions of the employed cementitious materials (%).

Compositions	Na_2_O	MgO	Al_2_O_3_	SiO_2_	P_2_O_5_	SO_3_	K_2_O	CaO	Fe_2_O_3_	LOI
Cement	0.09	1.61	4.18	19.20	0.09	3.35	0.78	64.93	3.32	2.49
SF	0.13	0.47	0.25	94.65	0.17	0.69	0.84	0.36	0.15	2.29
FA	0.33	0.23	38.01	46.44	0.06	0.69	0.88	7.50	3.12	2.79

**Table 2:** Recipe of the designed UHPC combined with damp pumice particles (kg/m^3^).

Group	Cement	FA	SF	River Sand		Damp Pumice		Water	SP
				0–0.6	0.6–1.25	0–0.6	0.6–1.25		
C0	750	200	144	770	220	0	0	175	31
UP_1_C10	750	200	144	693	220	37.08	0	175	31
UP_1_C20	750	200	144	616	220	74.16	0	175	31
UP_1_C30	750	200	144	539	220	111.24	0	175	31
UP_2_C10	750	200	144	770	198	0	6.00	175	31
UP_2_C20	750	200	144	770	176	0	12.00	175	31
UP_2_C30	750	200	144	770	154	0	18.00	175	31
SP_1_C10	750	200	144	693	220	55.43	0	175	31
SP_1_C20	750	200	144	616	220	110.86	0	175	31
SP_1_C30	750	200	144	539	220	166.29	0	175	31
SP_2_C10	750	200	144	770	198	0	8.38	175	31
SP_2_C20	750	200	144	770	176	0	16.76	175	31
SP_2_C30	750	200	144	770	154	0	25.14	175	31

**Table 3:** Fluidity of the designed UHPC with different amounts of extra water introduced by wet pumice.

Group	Extra Water	w/b		Fluidity (mm)
		Free	Total	
C0	0	0.1826	0.1826	183
UP_1_C10	5.9		0.1880	167
UP_1_C20	11.8		0.1934	173
UP_1_C30	17.7		0.1988	214
UP_2_C10	0.96		0.1835	165
UP_2_C20	1.92		0.1844	180
UP_2_C30	2.88		0.1853	201
SP_1_C10	24.3		0.2048	269
SP_1_C20	48.6		0.2271	345
SP_1_C30	72.9		0.2493	374
SP_2_C10	3.35		0.1857	147
SP_2_C20	6.7		0.1888	181
SP_2_C30	10.05		0.1918	186

**Table 4:** Generation of Ca(OH)_2_ for corresponding representative groups based on calculation by DTG curves (%).

Group	C0	UP_1_C30	SP_2_C10	SP_2_C30
7 d	0.387	0.517	0.506	0.550
28 d	0.420	0.348	0.385	0.423
